# Seasonal changes in the reproductive performance in local cows receiving artificial insemination in the Pursat province of Cambodia

**DOI:** 10.5713/ajas.19.0893

**Published:** 2020-04-12

**Authors:** Bengthay Tep, Yasuhiro Morita, Shuichi Matsuyama, Satoshi Ohkura, Naoko Inoue, Hiroko Tsukamura, Yoshihisa Uenoyama, Vutha Pheng

**Affiliations:** 1 Asian Satellite Campus in Cambodia, Nagoya University, c/o Royal University of Agriculture, Khan Dangkor, Phnom Penh, P.O. Box 2696, Cambodia; 2 Department of Animal Health and Veterinary Public Health, General Directorate of Animal Health and Production, Khan Meanchey, Phnom Penh, 12352, Cambodia; 3 Asian Satellite Campuses Institute, Nagoya University, Nagoya, Aichi, 464-8601, Japan; 4 Laboratory of Animal Production Science, Graduate School of Bioagricultural Sciences, Nagoya University, Nagoya, Aichi, 464-8601, Japan; 5 Laboratory of Animal Reproduction, Graduate School of Bioagricultural Sciences, Nagoya University, Nagoya, Aichi, 464-8601, Japan; 6 Prekleap National Institute of Agriculture, Khan Chhroychangva, Phnom Penh, 12112, Cambodia

**Keywords:** Artificial Insemination, Body Condition, Cambodia, Cows, Reproductive Performance, Seasonal Changes

## Abstract

**Objective:**

The present study aimed to survey seasonal changes in reproductive performance of local cows receiving artificial insemination (AI) in the Pursat province of Cambodia, a tropical country, to investigate if ambient conditions affect the reproductive performance of cows as to better understand the major problems regarding cattle production.

**Methods:**

The number of cows receiving AI, resultant number of calving, and calving rate were analyzed for those receiving the first AI from 2016 to 2017. The year was divided into three seasons: cool/dry (from November to February), hot/dry (from March to June), and wet (from July to October), based on the maximal temperature and rainfall in Pursat, to analyze the relationship between ambient conditions and the reproductive performance of cows. Body condition scores (BCS) and feeding schemes were also analyzed in these seasons.

**Results:**

The number of cows receiving AI was significantly higher in the cool/dry season than the wet season. The number of calving and calving rate were significantly higher in cows receiving AI in the cool/dry season compared with the hot/dry and wet seasons. The cows showed higher BCSs in the cool/dry season compared to the hot/dry and wet seasons probably due to the seasonal changes in the feeding schemes: these cows grazed on wild grasses in the cool/dry season but fed with a limited amount of grasses and straw in the hot/dry and wet seasons.

**Conclusion:**

The present study suggests that the low number of cows receiving AI, low number of calving, and low calving rate could be mainly due to poor body condition as a result of the poor feeding schemes during the hot/dry and wet seasons. The improvement of body condition by the refinement of feeding schemes may contribute to an increase in the reproductive performance in cows during the hot/dry and wet seasons in Cambodia.

## INTRODUCTION

Cambodia reportedly produced milk for domestic consumption and also exported it to neighboring countries in South East Asia, such as Thailand and Vietnam, before the civil war from the 1970s to 1990s [1]. As an unfortunate result of this civil war, the animal husbandry sector including the dairy industry was completely decimated and the country was forced to start from zero afterward. Currently, the total number of cattle in Cambodia is 2.95 million, of which more than 99% are reared by small-scale farmers in rural areas [2]. Keeping cows is a very common practice for these rural small-scale farmers, because cattle provide draft power for tillage and/or transportation, their waste serves as a raw material of fertilizer and biogas, and eventually the cattle themselves are a stock of wealth for conversion into cash for household expenses [3,4].

During the last 25 years, the Cambodian population has increased from 9.9 to 15.3 million [5], which has brought a significant increase in demand for food for consumption including animal products. The average meat consumption per person in Cambodia was reported to be 17.6 kg per year in 2017 [2] which was the lowest level in the world [6]. Previous studies suggested that Cambodians need more animal products as a source of protein to meet nutrient requirements [7,8]. Thus, an increase in animal production is crucially important in Cambodia. Significantly, an increase in cattle production would have great potential to improve the livelihood and well-being of the rural small-scale farmers who are still in poverty.

Reproductive performance of livestock is a key trait for animal production, since successful pregnancy and parturition directly increase productivity and profitability in animal production. The reproductive performance of local cows, such as the native yellow cattle (*Bos indicus*) and crossbreeds of the native yellow cattle and Hariana cattle (an Indian zebuine beef cattle, *Bos indicus*), however, is poorly understood in Cambodia. Previous studies reported that the age at the first calving and calving intervals were approximately 4 years and 14 to 20 months, respectively, in several provinces of Cambodia [9–13]. The reproductive performance of cows in Cambodia seems lower than other tropical Zebu cows, whose age at the first calving (2 to 5 years) and calving intervals (11 to 21 months) were reported in tropical Asian and African countries, such as Malaysia, Sri Lanka, and Kenya [14]. The poor reproductive performance of cows in Cambodia seems to be mainly due to poor nutrition and mating management because of a lack of farmer’s knowledge concerning animal nutrition and reproduction [3,11,12]. Specifically, most of the cattle were reared with wild grasses and rice straws, which has poor nutrition, and a limited amount of feed was also reported to be a common problem in cattle production in several provinces of Cambodia previously studied [3,13]. On the other hand, farmers seem to barely pay attention to the reproductive performance of cows, because cattle were mainly raised for draft power, not for beef or milk production in Cambodia. Besides, farmers used to hardly invest in their cattle, because the return for cattle production takes longer than that for crop and vegetable production [13]. More recently, Cambodian farmers are increasingly interested in beef cattle production because of the growing demand for animal products.

To improve beef cattle production in Cambodia, it is im portant to introduce artificial insemination (AI) and analyze the reproductive ability of cows receiving the AI. Pursat province is one of the suitable survey sites, because the AI was introduced in this province by the Program of Promotion of Inclusive and Sustainable Growth in the Agricultural Sector-Fisheries and Livestock—funded by the European Union—in 2015 to improve beef cattle production by using the semen of exotic breeds: Brahman breed (an American zebuine beef cattle, *Bos indicus*) and Indo-Brazil breed (a Brazilian zebuine beef cattle, *Bos indicus*). AI is not yet popular in other provinces in Cambodia and the majority of cows are still mated with local bulls, which were reared within the village or brought from neighboring villages to obtain calves, as reported previously [13,15].

The present study aims to investigate the reproductive per formance of local Cambodian cows receiving AI in Pursat province for a better understanding of the major problem of cattle production in Cambodia. We mainly focused on seasonal changes in the reproductive performance and body condition score (BCS) of cows under the tropical climate of Cambodia with its high temperature and humidity. The feeding schemes were also analyzed via interviews with farmers, since the seasonal feed availability has been known to largely affect the reproductive performance and body condition of cows.

## MATERIALS AND METHODS

### Study site, climate, and interview

**Figure 1:**
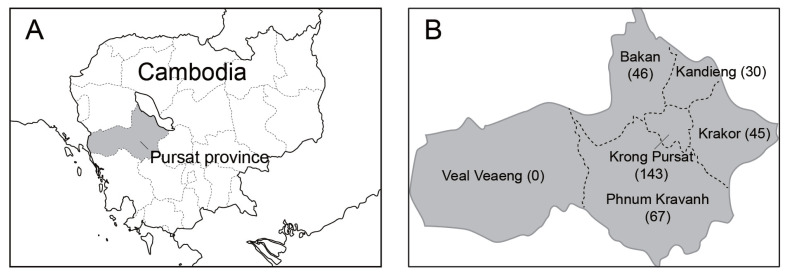
Map of the study sites. (A) The gray-shaded area surrounded by the dotted line shows the location of Pursat province in Cambodia. (B) The geographical distribution of surveyed cows at the district level. Numbers in parentheses indicate the numbers of cows analyzed. Note that the artificial insemination project was not yet expanded to Veal Veaeng district from 2016 to 2017. The map was downloaded from http://www.freemap.jp (accessed 6th June 2019) and modified.

The present survey was conducted in Bakan, Kandieng, Krakor, Phnom Kravanh, and Krong Pursat districts, Pursat province of Cambodia (Figure 1). A large number of cattle are kept in Pursat province (Table 1). The number of farms that participated in the current survey and the median and range of the number of cows and heifers as well as the total number of cattle reared by each farm are shown in Table 2. At the beginning of the current AI program, technicians of the Pursat Provincial Office of Animal Health and Production, informed the farmers about the advantages of the AI to improve beef cattle production. The farmers volunteered to participate in the AI program and paid the fee of AI. Sixty-five out of the 224 farms had participated in the previous AI program by the Small Livestock Production Program of the Food and Agriculture Organization—funded by the European Union— from 2007 to 2010.

**Figure 2:**
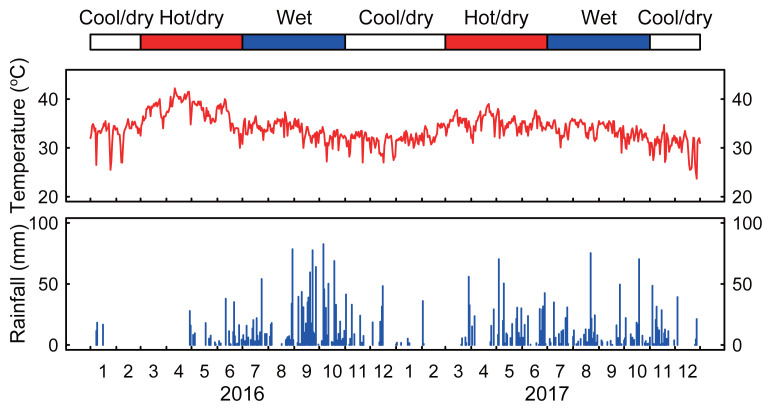
The maximal ambient temperature and amount of rainfall in Pursat province during 2016 and 2017. The data were obtained from the Provincial Department of Water Resource and Meteorology, Pursat.

The records of the maximal ambient temperature and rain fall in the province during 2016 and 2017 were obtained from the Provincial Department of Water Resource and Meteorology, Pursat. Raw data is shown in Figure 2. The year was divided into three seasons: cool/dry (from November to February), hot/dry (from March to June), and wet (from July to October). Mean and maximal temperature and the total rainfall during the three seasons are shown in Table 3.

Seasonal changes in land usage and availability of food for cows were analyzed by interviews with all the farmers in order to examine the feeding schemes of cows in this study site. The interviews were conducted by the first author and AI technicians.

### Analysis of the reproductive performance and body condition of cows receiving AI

The number of cows receiving AI, resultant number of calving, and calving rate were analyzed for those receiving the first AI from 2016 to 2017. The estrous behavior of local cows and heifers (3 years old or more, because heifers show puberty onset at approximately 3 years old)—native yellow cattle (*Bos indicus*) and crossbreeds of native yellow cattle and Hariana cattle (an Indian zebuine beef cattle, *Bos indicus*)—were monitored by farmers and confirmed by trained AI technicians of the Pursat Provincial Office of Animal Health and Production. The AI with frozen bull semen of Brahman or Indo-Brazil was performed by 4 trained technicians, 12 to 24 h after cows or heifers showed an obvious estrous behavior. Age, parity, and BCS of each cow were recorded at the timing of AI by the AI technicians. The resultant calving was also recorded by AI technicians. The calving rate was calculated by dividing the number of cows that delivered calves by the total number of cows and heifers receiving the AI during each season. The characteristics of local cows, such as coat colors, shape, and body sizes, are described elsewhere [9].

The BCS was evaluated in order to assess the nutritional status of each cow by using a 5-point scale with quarter-point divisions (1.0 to 5.0) according to Edmonson et al [16]. Scores 1.0 and 5.0 represent severe under conditioning and severe over conditioning, respectively and scores around 3.0 represents well-balanced conditioning for Holstein cows.

### Statistical analysis

The difference in the number of cows subjected to AI, the number of calving, and the resultant caving rate between seasons were analyzed by chi-square test with Bonferroni adjustment (R version 3.5.0, http://www.R-project.org/). The difference in the BCS of cows receiving AI between seasons were analyzed by the Kruskal-Wallis test followed by pairwise Wilcoxon rank sum test with Bonferroni adjustment (R version 3.5.0).

## RESULTS

### Number, age, and body condition scores of cows

**Figure 3:**
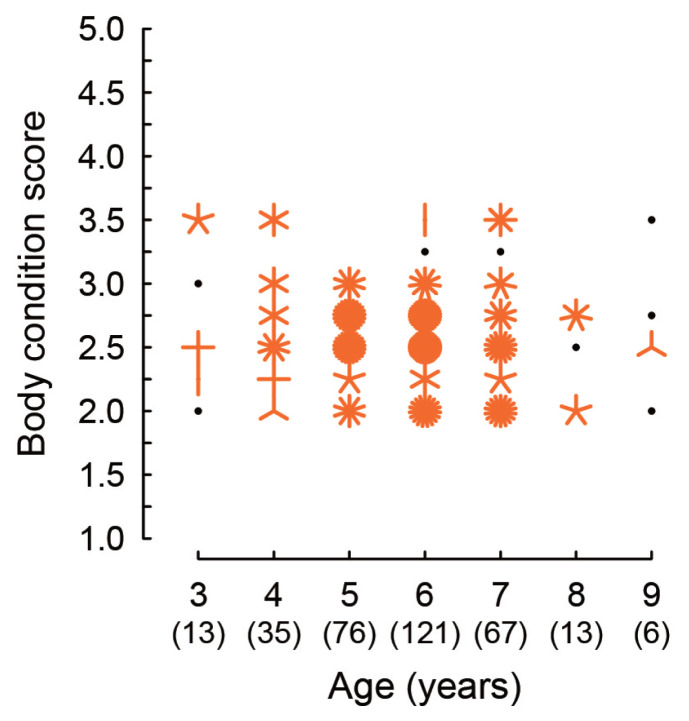
The body condition score of cows at the timing of artificial insemination. Black dots indicate that there is one cow observed at the coordinate value. When two or more cows were observed at the same coordinate value, the number of cows is expressed as the number of “petals” in orange. Numbers in parentheses of the X-axis indicate the numbers of cows evaluated in each age group.

Figure 3 shows the ages and the BCSs of cows subjected to AI in Pursat province from January 2016 to December 2017 as a sunflower plot. The numbers of cows counted in each age are shown in parentheses of the X-axis of Figure 3. The ages of cows ranged from 3 to 9 years old (6±1.2 years old on average) and a large number of cows were scored BCS 2.5 (n = 110), 2.75 (n = 83), or 2.0 (n = 53) at the timing of AI. No cows scored as less than 2.0 and more than 3.5 were found in the AI record.

### Reproductive performance

**Figure 4:**
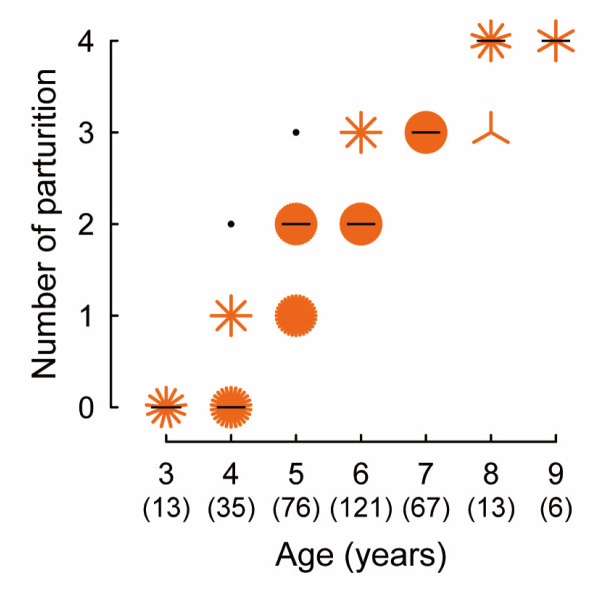
The number of parturitions recorded during the lifetime of each cow at the timing of artificial insemination. Black dots indicate that there is one cow observed at the coordinate value. When two or more cows were observed at the same coordinate value, the number of cows is expressed as the number of “petals” in orange. The median of the number of parturitions in each age are indicated by black horizontal bars. Numbers in parentheses of the X-axis indicate the numbers of cows evaluated in each age group.

**Figure 5:**
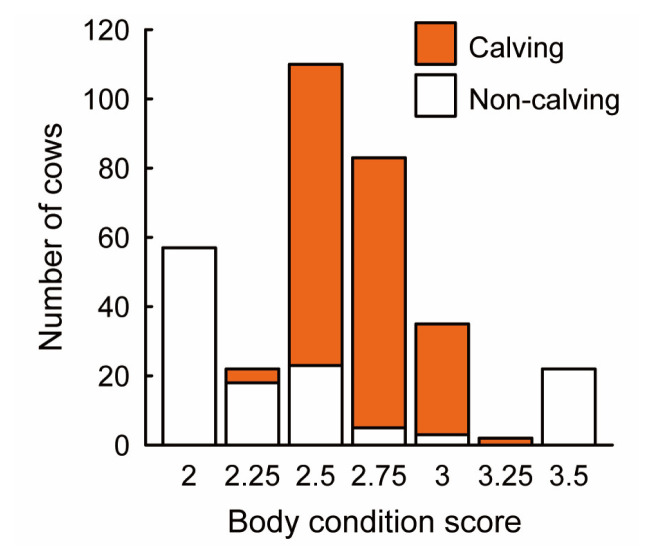
The proportion of cows that successfully delivered a calf in each the body condition score group. The closed and open bars indicate the number of the cows delivered a calf and failed to deliver, respectively.

The number of parturitions recorded for each cow during the lifetime is shown as a sunflower plot in Figure 4. The majority of cows showed the first parturition by the age of 4 years old. As the age of cows increased, the median of the parity increased by approximately one, between 4 to 8 years old. The relationship between the calving and BCS of cows is shown in Figure 5. The majority of cows, scored BCS 2.5 to 3.25 at the timing of AI, successfully delivered a calf. No cows who scored BCS 2.0 or 3.5 at the timing of AI, delivered a calf.

**Figure 6:**
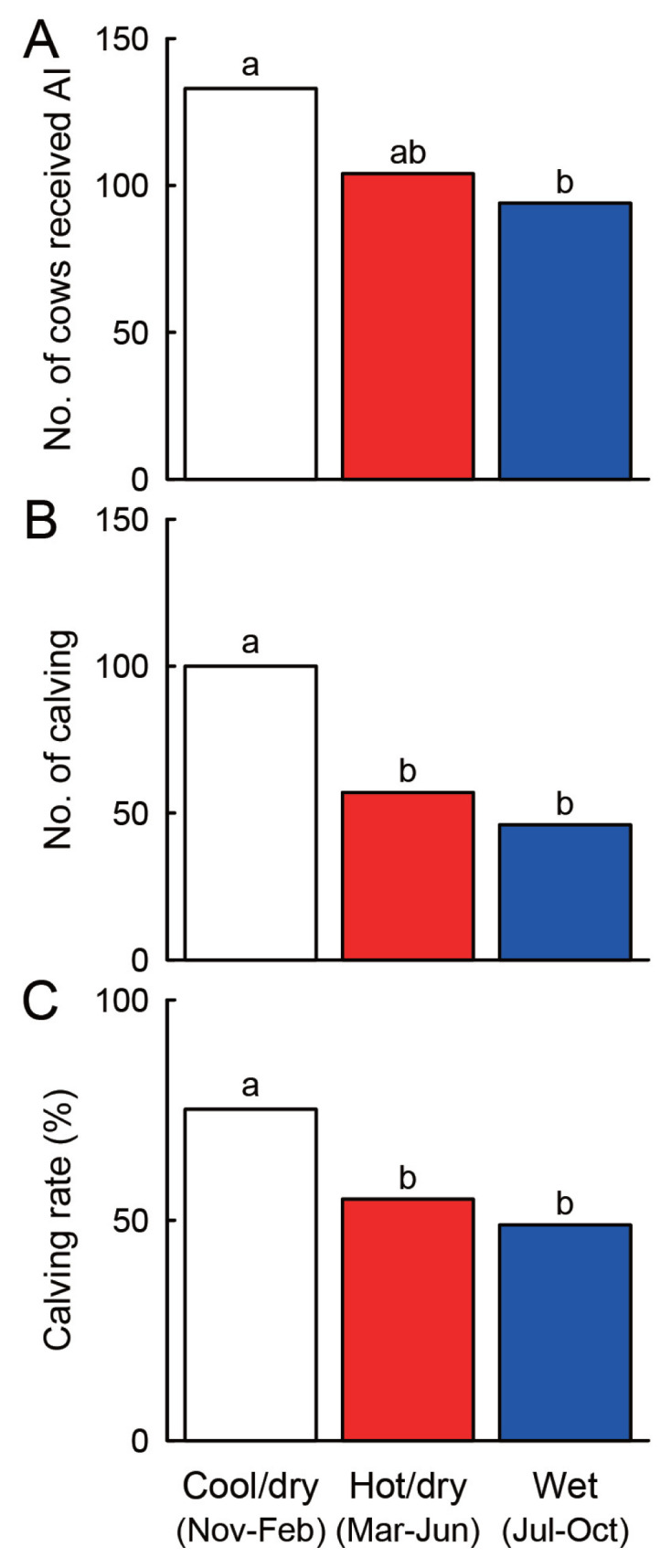
Seasonal changes in the number of cows receiving artificial insemination (A), the number of calving (B), and the resultant calving rate (C). Different letters indicate statistical significance (p<0.05, chi-square test with Bonferroni adjustment).

The number of cows receiving AI, the number of calving, and the resultant calving rate fluctuated from season to season. The number of cows receiving AI was significantly higher in the cool/dry season (from November to February) than the wet season (from July to October) (p<0.05, chi-square test with Bonferroni adjustment; Figure 6A). The number of calving and the calving rate were significantly higher in cows receiving AI in the cool/dry season compared with the hot/dry season (from March to June) and wet season (p<0.05, chi-square test with Bonferroni adjustment; Figures 6B and 6C).

### Feeding schemes

From November to February (after rice harvesting), cows mainly grazed on wild grasses in the rice field, which was covered with rice (*Oryza sativa*), *Brachiaria mutica*, *Digitaria ciliaris*, *Cyperus rotundus*, *Chrysopogon aciculatus*, *Eleusine indica*, *Cynodon dactylon*, and leguminous grasses. On the other hand, these wild grasses almost withered under the hot climate (from March to May) and then the rice field was occupied by the rice cultivation (from June to November). As a result, cows were fed a restricted amount of the aforementioned grasses, wild rice (*Oryza rufipogon*) and rice straw from March to October.

**Figure 7:**
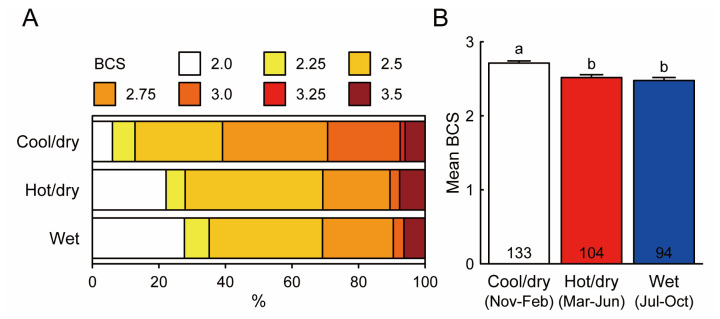
Seasonal changes in body condition score (BCS) of cows receiving artificial insemination (AI). The distribution of BCS (A) and mean BCS of cows receiving AI in three seasons (B). The data with different letters are statistically significant from each other (p<0.05, Kruskal-Wallis test followed by pairwise Wilcoxon rank sum test with Bonferroni adjustment). Numbers in the column indicate the numbers of cows receiving AI in each season.

Figure 7A shows the distribution of BCSs of cows in three seasons: 81.2% of cows were scored BCS 2.5 to 3.25, the majority of which successfully showed the delivery, during the cool/dry season, whereas 64.4% and 58.5% of cows were scored BCS 2.5 to 3.25 during the hot/dry season and the wet season, respectively. Cows exhibited significantly higher BCSs in the cool/dry season compared with the hot/dry and wet seasons (p<0.05, Kruskal-Wallis test followed by pairwise Wilcoxon rank sum test with Bonferroni adjustment; Figure 7B).

## DISCUSSION

The present study indicates seasonal changes in reproductive performance of local Cambodian cows: both the number of cows receiving AI, the number of calving, and the resultant calving rate were highest in the cool/dry season and decreased during the hot/dry and wet seasons. The BCS of cows receiving AI was also highest in the cool/dry season and decreased during the hot/dry and wet seasons, suggesting that the seasonal changes in reproductive performance of local cows are likely due to the seasonal changes in body condition of cows under a tropical climate in Cambodia. It should be noted that estrus detection and AI was performed by trained technicians of the Pursat Provincial Office of Animal Health and Production and no technical problem was observed, because the calving rate of cows receiving AI during the cool/dry season was sufficient (>75%), compared with previous studies showing 23% to 72% conception rates in Thailand [17] and Vietnam [18], indicating the AI was successfully performed by well-trained technicians in the current study. Taken together, these results suggest that the improvement of body condition may increase the reproductive performance in cows during the hot/dry and wet seasons in Cambodia.

It is well known that poor body condition exerts a nega tive influence on the reproductive performance of cows, such as delayed puberty and prolongation of postpartum anestrus [19,20]. Previous studies showed that increases in BCS resulted in the resumption of estrous cyclicity with increased concentration of circulating gonadotropins in anestrus cows [21] and heifers [22] in the United States. Thus, an improvement of the body condition is a key management strategy to increase the number of cows with regular estrous cycles during the hot/dry and wet seasons in Cambodia. Interestingly, fatty body condition also exerts a negative influence on the reproductive function of cows, because cows scored BCS 4 and higher had longer days from the delivery to the first AI and the conception [23]. Indeed, no cow scored BCS 3.75 or higher was found in the record for AI in the current study. Thus, the body condition of cows should be kept at BCS 2.0 to 3.5 for local Cambodian cows to improve their reproductive performance. Indeed, the present study showed that the cows receiving AI, namely cows having a regular estrous behavior, were scored BCS 2.0 to 3.5. Additionally, previous studies showed that poor body condition negatively affects oocyte competence [24]. Snijders et al [25] previously showed that *in vitro* fertilized oocytes of cows in lower body condition had a lower cleavage rate and a lower developing rate to the blastocyst stage compared with oocytes of cows in better body condition. Further, a previous study estimated 70% to 80% of embryonic loss occurred between day 8 and 16 after AI [26]. Taken together, the low calving rate of cows receiving AI during the hot/dry and wet seasons is likely due to early embryonic death, although causes of non-calving were not stated in the current record of AI.

It seems that the seasonal changes in the body condition of cows are mainly due to the seasonal changes of feeding schemes, because cows were fed with a limited amount of food during the hot/dry and wet seasons in Pursat province. Mob et al [13] previously showed a similar pattern of seasonal changes of feeding schemes (mainly dependent on wild grasses) and poor reproductive performance of cattle (calving interval was approximately 14 months) in Battambang and Pailin provinces, which are the provinces adjacent to the north of Pursat province, and reported that poor feeding was the second constraint of cattle production as an answer from farmers; the biggest constraint of cattle production was infectious diseases. Sath et al [9] also reported a similar poor feeding scheme (mainly dependent on wild grasses) and poor reproductive performance (calving interval was approximately 18 months) in Takeo province in southern Cambodia. The current results are consistent with those results obtained from the aforementioned studies in terms of the relationship between poor nutrition and reproduction [9,13]. Improvement of feeding schemes with forage and silage during the hot/dry and wet seasons is urgently needed. In this context, it is noteworthy that the forage plantation was established in some villages of southern Cambodia, where farmers had learned about cattle nutrition and forage cultivation [3,11]. Improvement of feeding schemes with local cereal crops is also promising to increase the reproductive performance of cows. Indeed, our recent study in Afghanistan, an arid country [27], showed that cows fed with only straw exhibited lower BCS and poor reproductive performance and that feeding with straw and local cereal crops, such as maize bran, cottonseed cake, wheat, and corn, successfully improved body condition as well as reproductive performance of cows. Heat stress under the high ambient temperature and humidity in Cambodia may also have caused severe physical impact to cows: in fact, our previous study in Cambodia revealed that direct sunlight during the hot/dry season caused an increase in sympathetic nervous activity, a stress response, in crossbred dairy cows [28]. Further, De Rensis and Scaramuzzi [29] suggested that reduced fertility under the summer heat stress in cows may be due to both hyperthermia and poor appetite. Taken together, the current results that the number of cows receiving AI decreased in the hot/dry and wet seasons imply that the low reproductive performance of cows in Cambodia would be due to poor nutrition and the heat stress in the hot/dry and wet seasons.

The present study indicates that farmers and AI techni cians in the current study manage relatively well the mature cows compared to other provinces, where poor calving intervals (1.5 years or more) were reported previously [9–12], because the majority of cows receiving AI in the present study were experienced parturition every year after the first calving at 4 years of age. The late-onset of puberty is a common characteristic of reproduction in *Bos indicus* breeds: previous studies demonstrated that the *Bos indicus* breeds grow slower than *Bos taurus* breeds and take 2 to 3 years to reach puberty onset [14,30]. The current results that the majority of heifers showed the first calving at 4 years might be due to poor feeding schemes during the growing period. The lifetime productivity in female livestock including cows largely depends on the timing of puberty onset as well as the duration of postpartum anestrus. Thus, improvement of the feeding schemes of heifers during growing as well as the postpartum period of cows may contribute to an improvement of the reproduction of cows in Cambodia.

In conclusion, the present study suggests that the low num ber of cows receiving AI and the low calving rate could be due to poor body condition as a result of the poor feeding schemes during the hot/dry and wet seasons. The improvement of the body condition by a refinement of these feeding schemes with forage and silage may contribute to an increase in the reproductive performance of cows during the hot/dry and wet seasons in Cambodia.

## Figures and Tables

**Table 1:** Cattle population in Pursat Province in 2016

Districts	Bulls	Others			

		<3 yrs old		≥3 yrs old	
	
		Male	Female	Male	Female
Bakan	131	6,554	7,072	4,538	7,131
Kandieng	27	1,715	2,044	1,893	2,324
Krakor	17	3,303	3,582	4,153	4,091
Phnom Kravanh	13	3,163	2,965	2,473	3,116
Krong Pursat	12	2,749	2,278	2,225	2,900
Veal Veaeng	25	1,671	1,844	1,256	1,456

The raw data was obtained from the Pursat Provincial Office of Animal Health and Production.

**Table 2:** The number of farms that participated in the current survey and the median and range of the number of cows and heifers as well as the total number of cattle reared by each farm

Districts	Farms	The number of cows and heifers (>3 yrs old)/fam		Total number of male and female cattle/farm
	
		Median	Range	Range
Bakan	33	2	1–8	2–17
Kandieng	25	2	1–4	1–6
Krakor	33	2	1–5	1–8
Phnom Kravanh	53	2	1–5	1–8
Krong Pursat	80	2	1–16	1–31

The raw data was obtained from the Pursat Provincial Office of Animal Health and Production.

**Table 3:** Mean and maximal ambient temperature and the total rainfall during the cool/dry, hot/dry, and wet seasons in Pursat province

Seasons	Temperature (mean [max, °C])1)	Rainfall (total, mm)1)
Cool/dry season (from November to February)	33.2 (35.9)	314.05
Hot/dry season (from March to June)	37.6 (42.2)	465.95
Wet season (from July to October)	35.5 (37.3)	946.90

1) The raw data were obtained from the Provincial Department of Water Resource and Meteorology, Pursat.
